# Role of liver augmentation prior to hepatic resection – a survey on standards, procedures, and indications in Germany, Switzerland, and Austria

**DOI:** 10.1007/s00423-024-03418-5

**Published:** 2024-07-27

**Authors:** Elif Yilmaz, Giovanni F. Torsello, Ali Seif Amir Hosseini, Anne-Christine Zygmunt, Thomas Lorf, Jan Keck, Stina Schild-Suhren, Björn Wellge, Rupert Oberhuber, Otto Kollmar, Michael Ghadimi, Florian Bösch

**Affiliations:** 1 Department of General, Visceral, and Pediatric Surgery, University Medical Center Göttingen (UMG), Robert-Koch-Straße 40, 37075 Göttingen, Germany; 2 Department of Diagnostic and Interventional Radiology, University Medical Center Göttingen, Göttingen, Germany; 3 Department of Visceral, Transplant and Thoracic Surgery, Center for Operative Medicine, Medical University of Innsbruck, Innsbruck, Austria; 4 Department of Visceral Surgery, University Center for Gastrointestinal and Liver Diseases, St. Clara Hospital and University Hospital, Basel, Switzerland

**Keywords:** Liver augmentation, Future liver remnant, Portal vein embolization, Associating liver partition and portal vein occlusion for staged hepatectomy

## Abstract

**Purpose:**

For primary and secondary liver tumors oncological resection remains a chance of cure. Augmentation of functional liver tissue may be necessary to preserve sufficient future liver remnant (FLR). Clinical decision-making on liver augmentation techniques and indications may differ internationally. Thus, this study aims to identify standards of liver augmentation in hepato-pancreatico-biliary (HPB) centers in Germany, Switzerland, and Austria.

**Methods:**

Using a web-based survey, 48 hospitals in Germany, Switzerland, and Austria were invited to report their surgical indication, standard procedures, and results of liver augmentation.

**Results:**

Forty (83.3%) of the hospitals invited participated. Most of the hospitals were certified liver centers (55%), performing complex surgeries such as liver transplantation (57.5%) and ALPPS (80%). The standard liver augmentation technique in all countries was portal vein embolization (PVE; 56%), followed by ALPPS (32.1%) in Germany or PVE with hepatic vein embolization (33.3%) in Switzerland and Austria. Standard procedure for liver augmentation did not correlate with certification as liver center, performance of liver transplantation or ALPPS. Surgical indication for PVE varied depending on tumor entity. Most hospitals rated the importance of PVE before resection of cholangiocarcinoma or colorectal metastases as high, while PVE for hepatocellular carcinoma was rated as low.

**Conclusion:**

The survey gives an overview of the clinical routine in HPB centers in Germany, Austria, and Switzerland. PVE seems to dominate as standard technique to increase the FLR. However, there is a variety in the main indication for liver augmentation. Further studies are necessary evaluating the differing PVE techniques for liver augmentation.

**Supplementary Information:**

The online version contains supplementary material available at 10.1007/s00423-024-03418-5.

## Introduction

Future liver remnant (FLR) limits the indication for oncological liver resection due to the risk of posthepatectomy liver failure. Adequately measuring FLR prior to resection is consequently necessary to avoid postoperative liver failure. However, to evaluate the risk of post-hepatectomy liver failure not only size but also function of the remaining tissue must be considered. Therefore, it might be necessary to assess the growth rate of the FLR or use liver function tests such as LiMON [1, 2]. Primary liver tumors may develop in a cirrhotic liver, whereas patients with secondary liver metastases, i.e. colorectal liver metastases (CRLM) [3, 4], undergo chemotherapy prior liver resection and suffer from chemotherapy-induced liver injury [5]. Severely deteriorated liver tissue due to chemotherapy or cirrhosis increases the risk for post-hepatectomy liver failure. While an oncological resection of malignant liver tumors allows a curative therapy option, the ‘too-small-for-size’ liver is therefore one of the leading reasons for high morbidity following major liver resection [6–9].

Several liver augmentation techniques have been discussed for optimal hypertrophy results in case of insufficient FLR. An interventional approach by embolization of the portal vein (PVE) induces hypertrophy of the FLR by approximately 30–50% within four to eight weeks, with a resectability rate of up to 75% and considerably low morbidity [10–12]. If necessary, additional segment IV embolization and/or hepatic vein embolization (HVE) further increases and accelerates growth [13]. Complications through non-target-embolization are usually qualitatively mild and rare [12]. A surgical approach through associating liver partition and portal vein occlusion for staged hepatectomy (ALPPS) was demonstrated as a fast and strategically effective technique to increase liver tissue up to 74% in approximately ten days [14] with a 92% resectability rate but in comparison to PVE with a higher morbidity [15–17]. Furthermore, selective internal radiation therapy (SIRT), though not commonly used, is another technique to induce liver hypertrophy [18, 19].

Although liver augmentation techniques sufficiently induce hypertrophy, two staged hepatectomies (TSH) still might be challenging harboring considerable morbidity rates [12]. Depending on the liver augmentation technique, hypertrophy rate in relation to complications and surgical outcome differ. In this context, in previous literature it is not quite clear which technique should be used in the different indications for surgery. Also, a surgeon’s expertise and a hospital setting’ influence the approach of oncological resections. Therefore, clinical decision-making concerning liver augmentation techniques may differ in hepato-pancreatico-biliary (HPB) centers, but ensuring good clinical practice management strategies are needed. Thus, this study aims to identify clinical routine strategies of liver augmentation in HPB centers in Germany, Switzerland, and Austria.

## Materials and methods

By sending a web-based survey, 48 HPB centers in Germany, Switzerland, and Austria were invited to participate. Where possible, the questions were designed as closed questions to facilitate analysis. Other questions were designed to give the respondent options and we used the Likert scale to weight answers if necessary [20]. The questions were designed, reviewed, and validated by nine board certified hepatobiliary surgeons from Germany, Switzerland, and Austria and two board certified radiologists with a specialization in interventional radiology from Germany. The survey was designed from October to December 2022 in regular online meetings. Finally, the invitation to the questionnaire was sent by email and the participating surgeons have been given three months to complete it.

Either the head of the department or the section leader was contacted to ensure high quality. Generating a homogeneous group only university hospitals were invited in Germany and Austria. Due to the different health care system in Switzerland, non-university hospitals were also invited to participate. To maintain high quality statements, centers that met the following criteria were selected: university hospital and/or performing complex liver surgeries.

The invited hospitals in Germany and Austria were exclusively university hospitals and in Switzerland the leading HPB hospitals were invited to participate. All German hospitals were “high-volume” centers for liver surgery according to Filmann et al. [21]. Participation and consent to participate was carried out by answering the survey online via google forms. Participation was anonymous.

The survey contained 24 questions assessing the participating hospital’s volume and complexity of liver resections, clinical decision-making concerning surgical indication, used standard procedure, technical issues, and results of liver augmentation techniques depending on the underlying disease, since surgical and indication for liver augmentation might differ. Questions were either ordinal or nominal minded. The listed questions are shown in Appendix 1. Results were summarized and analyzed in categories and for the different countries. For the analysis of the collected data, descriptive statistics were employed to summarize the responses, thereby illustrating the trends and practices in liver surgery across Germany, Switzerland, and Austria. All data were analyzed using Microsoft Excel (Microsoft Corporation, Redmond, VA, USA) and GraphPad Prism (version 8.0, GraphPad Software Inc., Boston, MA, USA).

## Results

Forty of 48 (83.3%) hospitals invited participated. The attendance quota was 28/31 (90.3%) in Germany (G), 9/14 (64.2%) in Switzerland (S), and 3/3 (100%) in Austria (A). Most of the participating hospitals were certified liver centers (*n* = 22/40, 55%) and performed complex surgeries such as ALPPS (*n* = 32/40, 80%) or liver transplantation (*n* = 23/40, 57.5%). In Germany and Austria, most hospitals performed more than 100 liver resections per year (G *n* = 22/28, 78.5%; A *n* = 2/3, 66.7%) whereas in Switzerland all participating hospitals performed less than 100 liver resections. Since Switzerland has only a tenth of the population compared to Germany, these numbers are comparable. In Germany and Austria only university hospitals participated while in Switzerland 44.4% (4/9) were university hospitals (Table 1).

**Table 1:** Characteristics of participating centers in Germany, Switzerland, and Austria. Values in parenthesis are percentages (DKG, Deutsche Krebsgesellschaft – German Cancer society; DGAV, Deutsche Gesellschaft für Allgemein- und Viszeralchirurgie – German society for general and visceral surgery; ALPPS, associating liver partition and portal vein ligation for staged hepatectomy)

		Germany (*n* = 28)	Switzerland (*n* = 9)	Austria (*n* = 3)
University hospital	Yes	28 (100)	4 (44.4)	3 (100)
	No	0	5 (55.6)	0
Certified liver center (DKG, DGAV, etc.)	Yes	19 (67.9)	3 (33.3)	0 (0)
	No	9 (32.1)	6 (66.7)	3 (100)
Liver transplant program	Yes	17 (60.7)	3 (33.3)	3 (100)
	No	11 (39.3)	6 (66.7)	0
ALPPS performed	Yes	26 (92.9)	4 (44.4)	2 (66.7)
	No	2 (7.1)	5 (55.6)	1 (33.3)
Liver resections per year	≤100	6 (21.4)	9 (100)	1 (33.3)
	101–150	10 (35.7)	0	1 (33.3)
	151–200	6 (21.4)	0	1 (33.3)
	≥201	6 (21.4)	0	0
Portal vein embolizations per year	≤10	14 (50)	8 (88.9)	1 (33.3)
	11–20	7 (25)	1 (11.1)	1 (33.3)
	21–30	7 (25)	0	1 (33.3)

**Fig. 1:**
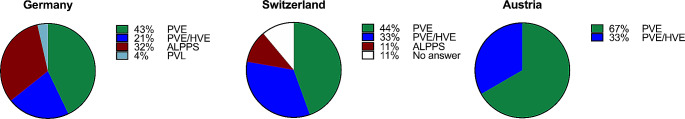
Standard augmentation technique used in participating centers in every country (PVE, portal vein embolization; PVE/HVE, portal and hepatic vein embolization; ALPPS, associating liver partition and portal vein ligation for staged hepatectomy; PVL, portal vein ligation)

The most common augmentation technique was PVE (G *n* = 12/28, 42.9%; S *n* = 4/9, 44.4%; A *n* = 2/3, 66.7%) in all countries, followed by ALPPS in Germany (*n* = 9/28, 32.1%), and PVE/HVE in Switzerland and Austria (S *n* = 3/9, 33.3%; A *n* = 1/3, 33.3%) (Fig. 1). We asked for possible reasons influencing the different choices for augmentation techniques since the chosen liver augmentation technique may be influenced by the number of performed liver resections. Nonetheless, the respective chosen standard procedure for liver augmentation did not correlate with the number of liver resections performed per year in any country.

A hospital setting may also influence the chosen augmentation technique. Thus, we analyzed whether the chosen augmentation technique differed between certified or non-certified hospitals. Certified liver centers predominantly chose PVE (*n* = 8/22, 36.4%), then ALPPS (*n* = 7/22, 31.8%) and PVE/HVE (*n* = 6/22, 27.3%), and portal vein ligation (PVL, *n* = 1/22, 4.5%) respectively. Non-certified hospitals also preferred PVE (*n* = 10/18, 55.6%), then PVE/HVE (*n* = 4/18, 22.2%) and ALPPS (*n* = 3/18, 16.7%) for liver augmentation, showing that certification had no influence. The vast majority of hospitals perform ALPPS (*n* = 32/40, 80%), but ALPPS is not the standard technique to induce hypertrophy. Within the clinics preforming ALPPS, PVE dominated as a standard (*n* = 13/32, 40.6%), followed by ALPPS (*n* = 10/32, 31.3%), then by PVE/HVE (*n* = 7/32, 21.9%).

**Fig. 2:**
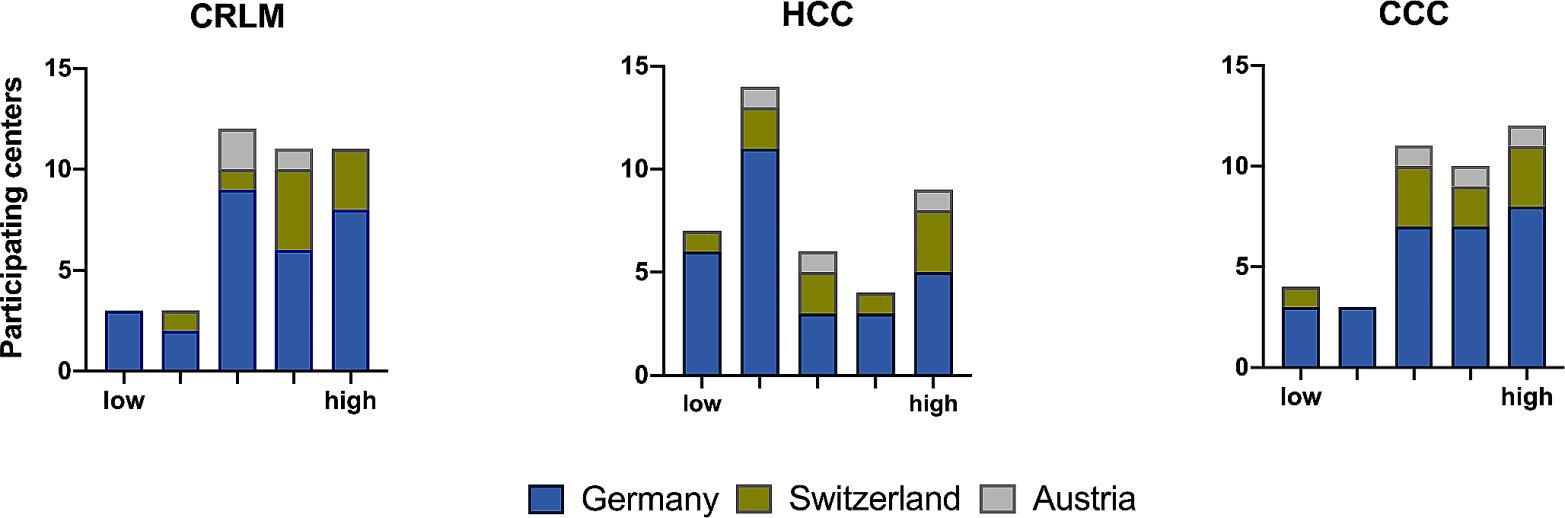
Level of importance (low to high) of PVE for the surgical therapy of colorectal liver metastases (CRLM, left), hepatocellular carcinoma (HCC, center) and cholangiocarcinoma (CCC, right)

Moreover, we also asked whether the underlying disease influences the indication for PVE (Fig. 2). A majority of 22 centers (55%) considered colorectal liver metastases (CRLM) and cholangiocarcinoma (CCC) to be a very good indication for PVE. In contrast, the majority of 21 centers (52.5%) considered hepatocellular carcinoma (HCC) to be a very poor indication for PVE. Nonetheless, still most participants through all countries saw an indication for hypertrophy in HCC with CHILD A cirrhosis (G *n* = 20/28, 71.4%; S *n* = 5/9, 55.6%; A *n* = 2/3, 66.7%). This study shows that the majority of participants experience SIRT neither as an adequate (G *n* = 18/28, 64.3%; S *n* = 5/9, 55.5%; A *n* = 2/3, 66.7%) nor a common technique (G *n* = 22/28, 78.6%; S *n* = 7/9, 77.8%; A *n* = 3/3, 100%) to induce hypertrophy.

**Fig. 3:**
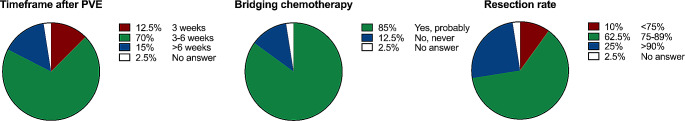
Handling of patients after PVE: After PVE most of the centers wait 3–6 weeks to resect (left) and the majority considered bridging chemotherapy (center). The resection rate after PVE was predominantly > 75% (right)

The majority of the hospitals used particles with coils/plugs to embolize the right branch of the portal vein (G *n* = 16/28, 57.1%; S *n* = 5/9, 55.6%; A *n* = 2/3, 66.7%). Complications due to non-target-embolization after PVE were rarely seen (all countries, *n* = 35/40, 87.5%), confirming PVE as safe. If FLR was considered insufficient after PVE, additional HVE might be performed [12, 13]. However, in Germany and Switzerland 32.1% (*n* = 9/28) and 44.4% (*n* = 4/9), respectively, never combined PVE and HVE. However, if PVE/HVE was done, it was mostly performed simultaneously (G *n* = 11/28, 39.3%; S *n* = 5/9, 55.6%; A *n* = 2/3, 66.7%). Most respondents through all countries at least considered additionally embolizing segment IV (G *n* = 19/28, 67.9%; S *n* = 8/9, 88.9%; A *n* = 2/3, 66.7%). Commonly the centers waited for 3–6 weeks for resection after PVE (*n* = 28/40, 70%) and during this period 85% of the hospitals (*n* = 34/40) treated patients with bridging chemotherapy (Fig. 3).

In Germany, 23 of 28 hospitals (82.1%) had a resection rate above 75% for embolized patients, while in Switzerland and Austria, all hospitals (*n* = 12/12; 100%) reported a resection rate above 75% of the embolized patients (Fig. 3). Progression of the hepatic tumor load was the main reason for no hepatic resection (G *n* = 16/28, 57.1%; S *n* = 3/9, 33.3%; A *n* = 1/3, 33%) followed by an insufficient hypertrophy of FLR (G *n* = 8/28, 28.6%; S *n* = 4/9, 44.4%; A *n* = 2/3, 66.7%). Assessment of liver function was not done on a regular basis. However, 42,9% (*n* = 12/28) in Germany, 33,3% (*n* = 3/9) in Switzerland and even 66,7% (*n* = 2/3) in Austria performed a liver function test if necessary.

## Discussion

The survey emphasizes the daily clinical routine of liver augmentation techniques in HPB centers in Germany, Switzerland, and Austria. The present study shows that PVE dominates as a technique to increase FLR, as reported in a recent minireview [22]. However, there is a wide variety in main indications for liver augmentation, particularly for PVE. With 40 contributing hospitals as centers for hepatobiliary surgery, the results of this overview are representative.

FLR has been shown as a major predictor among blood transfusion, hepatic parenchyma quality, and BMI of posthepatectomy liver failure [1, 13, 23]. To avoid post-hepatectomy liver failure because of insufficient FLR, which is considered the main cause of mortality after major liver resection [24], at minimum a ratio of FLR to body weight of 0.5 is needed [8, 9]. FLR values of > 20% of total liver volume with normal liver function [23, 25] and FLR > 30% in steatosis/hepatitis or > 40% in cirrhosis were defined as sufficient, respectively [8, 26, 27]. However, not only size, but also the degree of hypertrophy with a cut off value of < 5% correlated with posthepatectomy liver failure [25, 28], implying that not only the absolute amount of liver tissue but also the function and its ability to regenerate were crucial. A recent study showed that perioperative testing of liver function significantly reduced the complication rate [29]. In this respect, the recently published E-AHPBA–ESSO–ESSR Innsbruck consensus guidelines strongly recommend to assess the liver function prior to any liver augmentation strategy. Liver function tests should help to assess the discordance between the quantitative increase after augmentation and the real liver function [30]. However, the present survey revealed that assessment of liver function is not tested regularly. Only a third of the participating hospitals assess liver function on a regular basis. The present results also support this claim of rare testing, meanwhile showing that a low but considerable number of patients were not resected due to insufficient FLR after PVE. This births the question why testing was not frequently done beforehand, especially when functional liver measurement preoperatively may impact the decision making on augmentation. Additionally, our results confirm earlier studies highlighting the need for a standardized algorithm for liver augmentation techniques [31, 32].

Induction of hypertrophy has a significant impact on the long-term survival of patients with malignant liver tumors. In this respect, the prognosis of CRLM improved after introduction of liver augmentation techniques due to higher resection rates [15, 33, 34]. Especially for large liver metastases, surgery has better long-term results than other treatment options, such as chemotherapy alone or radiofrequency ablation [35]. Given that, one might expect that liver augmentation is needed. In clinical practice, the indication for PVE was ranked high for the surgical treatment of CRLM and was mostly chosen as a liver augmentation technique in hospitals in this study. Also, when PVE was performed, a high number of patients were resected thereafter, thus confirming earlier results of high resection rates [34].

On the one hand, PVE is a relatively simple intervention and has a comparatively low complication rate. Furthermore, it has only few contraindications, such as tumor invasion of the ipsilateral portal vein [36], making it a standard procedure to induce hypertrophy [13]. Additionally, to current recommendations for metastatic liver tumors, our study shows that PVE was also used for primary liver tumors, such as CCC or even HCC in CHILD A cirrhosis, making it a treatment option for a broad spectrum of diseases.

On the other hand, kinetic growth rate is relatively low and may take inadequately long periods of time considering the underlying oncological disease that is for this period left untreated. Consequently, augmentation of PVE by embolization the hepatic vein and segment IV have been suggested to allow a faster increase for two-staged hepatectomy [12, 13]. To analyze whether PVE/HVE accelerates further growth, the recruiting DRAGON 2 trial compares PVE to PVE/HVE in a randomized controlled trial [37]. In this study, additional HVE and segment IV embolization was also carried out or at least considered in most cases. It has been speculated that combining PVE and HVE might replace PVE in the near future [22]. During the time of growth, most hospitals considered treating patients with chemotherapy addressing the oncological needs.

The increase of the FLR depends on the embolisate used and a possible reflow in the embolized portal branch may decrease the hypertrophy effect [10, 38]. In this study, favored materials were particles with coils and plugs, while acrylic glue or mono-particles were significantly less frequently chosen.

In recent studies ALPPS was predominantly reserved for treating CRLM and has been shown to have a better long term survival in patients with CRLM compared to TSH after PVE [15, 16]. However, in the present analysis ALPPS was the preferred liver augmentation technique in only 31.3% (*n* = 10/32), although most of the hospitals performed a high number of major liver resections annually, including liver transplantation. This is in line with a former survey from 2015, however, PVE was chosen most likely in 35.2% to induce liver hypertrophy [39]. In this regard, in the present survey 56% see PVE as the standard approach for liver augmentation. Interestingly, only 5.6% of respondents then considered ALPPS to be a safe procedure whereas PVE was seen as safe in 81.5% [39]. Furthermore, the current survey did not take the hepatic tumor load or the distribution of CRLM into account. The indication for PVE or ALPPS might depend on the distribution of CRLM in the case of bilateral disease. If surgical clearing of the FLR in a first step is necessary, the subsequent augmentation technique might depend on the hepatic tumor load and the surgical approach. This issue has to be addressed in future studies. Another reason ALPPS may be less prevalent in clinical use might be the historically high morbidity and mortality rate after ALPPS as mentioned above. This however was due to a number of perioperative issues of the then newly introduced technique, unsuitable patient-selection, low surgical expertise, and postoperative care [31]. The ALPPS-registry was initiated to enable standardization by collecting data and identifying adequate indications for this procedure [31]. Although 80% of questioned hospitals performed ALPPS, our study shows that it was only the second choice for liver augmentation in Germany, third choice in Switzerland and no standard at all in Austria. Clinical standard for liver augmentation did not correlate with whether a hospital performed a high number of liver resections, was a certified liver center, or performed liver transplantation and ALPPS. In this study, the hospitals using ALPPS as a standard augmentation technique usually tested liver function prior to resection and mainly named insufficient FLR after PVE as reason to not resect – indicating that patient selection and anticipation of failure due to PVE might have led to the decision of operating firsthand.

Earlier studies demonstrated that SIRT can induce hypertrophy. Although SIRT showed lower growth rates and significantly longer growth periods compared to PVE [24, 40], it is an option when simultaneous treatment of the hepatic tumor burden is needed [19, 38]. A major disadvantage of PVE is an increased arterial flow of the tumor bearing lobe after portal obstruction which might support tumor growth during time of hypertrophy [41]. A radioembolization might bypass this by simultaneously damaging the tumor beforehand [42]. A recent study by Addeo et al. has shown in five patients that sequential PVE and yttrium-90 (^90^Y) liver radioembolization (TARE) can induce liver hypertrophy while downstaging liver tumors [37, 43]. Nonetheless, most of the hospitals did not experience SIRT as an adequate or common technique to induce hypertrophy. With proven success to induce hypertrophy, the European SIR-Spheres Surgical Registry (ESSURE) was introduced to improve comprehensibility of cases and optimize the process by recruiting patients [44]. Despite being a novel technique with lower growth rates, SIRT may still play a small role in liver augmentation [41, 42].

Our study has limitations. Methodically, the survey allowed mostly university hospitals to participate, possibly not representing the heterogeneity of hospitals treating primary and secondary liver tumors. However, with a considerable high number of participating hospitals and a high response rate the survey thus provides an insight into the healthcare landscape of German and Austrian university hospitals as well as a large part of Switzerland. Nonetheless, the present results need to be validated in a larger cohort of HPB surgeons not only in university hospitals. Also, a survey, phrasing general questions cannot predict a matter of consequence and answers given were surgeons opinions, which cannot exclude reporting bias. On the other hand, a survey is suitable to enable a general overview of the clinical routine. This study asked for the decision-making according to common choice, which naturally excludes generalization to all decision making and alters the results.

## Conclusion

In conclusion, PVE dominates as the standard liver augmentation technique in Germany, Austria, and Switzerland. Since postoperative complications after major hepatectomies and two-staged hepatectomies are associated with decreased long-term survival, this study underlines the need for further studies implementing indication algorithms for liver augmentation.

### Electronic supplementary material

Below is the link to the electronic supplementary material.

Supplementary Material 1

## Data Availability

No datasets were generated or analysed during the current study.

## Abbreviations

A: Austria
ALPPS: Associating liver partition and portal vein occlusion for staged hepatectomy
CCC: Cholangiocarcinoma
CRLM: Colorectal liver metastases
DGAV: Deutsche Gesellschaft für Allgemein- und Viszeralchirurgie,
German: Society for General and Visceral Surgery
DKG: Deutsche Krebsgesellschaft, German Cancer Society
ESSURE: European SIR-Spheres Surgical Registry
FLR: Future liver remnant
G: Germany
HPB: Hepato-pancreatico-biliary center
HCC: Hepatocellular carcinoma
HVE: Hepatic vein embolization
PVE/HVE: Portal and hepatic vein embolization
PVE: Portal vein embolization
PVL: Portal vein ligation
S: Switzerland
SIRT: Selective internal radiation therapy
TARE: Transarterial radioembolization
TSH: Two staged hepatectomy
