# Supplementary material for: Role of liver augmentation prior to hepatic resection – a survey on standards, procedures, and indications in Germany, Switzerland, and Austria
Source: Langenbecks Arch Surg. 2024 Jul 27;409(1):228. doi: 10.1007/s00423-024-03418-5 (PMC11283428; doi:10.1007/s00423-024-03418-5)
Supplement: Supplementary file 1 — Supplementary Material 1 [file 423_2024_3418_MOESM1_ESM.docx]

| Survey questions  Results presented in absolute numbers and percentages. | | Answer | Germany  (n=28) | Switzerland  (n=9) | Austria  (n=3) |
| --- | --- | --- | --- | --- | --- |
| 1 | What is the name of the hospital? |  |  |  |  |
| 2 | Is the hospital a certified liver center (DKG, DGAV, etc.)? | Yes | 19 (67.9) | 3 (33.3) | 0 |
|  |  | No | 9 (32.1) | 6 (66.7) | 3 (100) |
| 3 | Do you have a liver transplant program? | Yes | 17 (60.7) | 3 (33.3) | 3 (100) |
|  |  | No | 11 (39.3) | 6 (66.7) | 0 |
| 4 | Do you perform ALPPS? | Yes | 26 (92.9) | 4 (44.4) | 2 (66.7) |
|  |  | No | 2 (7.1) | 5 (55.6) | 1 (33.3) |
| 5 | How many liver resections are performed per year? | ≤100 | 6 (21.4) | 9 (100) | 1 (33.3) |
|  |  | 101-150 | 10 (35.7) | 0 | 1 (33.3) |
|  |  | 151-200 | 6 (21.4) | 0 | 1 (33.3) |
|  |  | ≥201 | 6 (21.4) | 0 | 0 |
| 6 | How many portal vein embolizations are performed per year? | ≤10 | 14 (50) | 8 (88.9) | 1 (33.3) |
|  |  | 11-20 | 7 (25) | 1 (11.1) | 1 (33.3) |
|  |  | 21-30 | 7 (25) | 0 | 1 (33.3) |
|  |  | 31-40 | 0 | 0 | 0 |
|  |  | ≥41 | 0 | 0 | 0 |
| 7 | What is your standard augmentation technique or most used technique to induce hypertrophy? | ALPPS | 9 (32.1) | 1 (11.1) | 0 |
|  |  | PVE | 12 (42.9) | 4 (44.4) | 2 (66.7) |
|  |  | PVL | 1 (3.5) | 0 | 0 |
|  |  | PVE/HVE | 6 (21.4) | 3 (33.3) | 1 (33.3) |
|  |  | SIRT | 0 | 0 | 0 |
|  |  | No answer | 0 | 1 (11.1) | 0 |
| 8 | How important do you reckon PVE for the surgical therapy of CRLM? | 1 | 3 (10.7) | 0 | 0 |
|  |  | 2 | 2 (7.1) | 1 (11.1) | 0 |
|  |  | 3 | 9 (32.1) | 1 (11.1) | 2 (66.7) |
|  |  | 4 | 6 (21.4) | 4 (44.4) | 1 (33.3) |
|  |  | 5 | 8 (28.6) | 3 (33.3) | 0 |
| 9 | How important do you reckon PVE for the surgical therapy of HCC? | 1 | 6 (21.4) | 1 (11.1) | 0 |
|  |  | 2 | 11 (39.3) | 2 (22.2) | 1 (33.3) |
|  |  | 3 | 3 (10.7) | 2 (22.2) | 1 (33.3) |
|  |  | 4 | 3 (10.7) | 1 (11.1) | 0 |
|  |  | 5 | 5 (17.9) | 3 (33.3) | 1 (33.3) |
| 10 | How important do you reckon PVE for the surgical therapy of CCC? | 1 | 3 (10.7) | 1 (11.1) | 0 |
|  |  | 2 | 3 (10.7) | 0 | 0 |
|  |  | 3 | 7 (25) | 3 (33.3) | 1 (33.3) |
|  |  | 4 | 7 (25) | 2 (22.2) | 1 (33.3) |
|  |  | 5 | 8 (28.6) | 3 (33.3) | 1 (33.3) |
| 11 | Do you see an indication to hypertrophy HCC in cirrhosis? If yes, which patients would you still consider for inducing hypertrophy? | No | 7 (25) | 3 (33.3) | 1 (33.3) |
|  |  | Child A | 20 (71.4) | 5 (55.6) | 2 (66.7) |
|  |  | Child B | 1 (3.6) | 0 | 0 |
|  |  | Child C | 0 | 0 | 0 |
|  |  | No answer | 0 | 1 (11.1) | 0 |
| 12 | Which embolization material are most used in your hospital? | Particles with coils/plugs | 16 (57.1) | 5 (55.6) | 2 (66.7) |
|  |  | Particles | 3 (10.7) | 2 (22.2) | 0 |
|  |  | Acrylic glue | 8 (28.6) | 2 (22.2) | 1 (33.3) |
|  |  | No answer | 1 (3.6) | 0 | 0 |
| 13 | Do you combine PVE with HVE? | Yes, sometimes | 18 (64.3) | 3 (33.3) | 2 (66.7) |
|  |  | Yes, always | 1 (3.6) | 2 (22.2) | 1 (33.3) |
|  |  | No | 9 (32.1) | 4 (44.4) | 0 |
| 14 | If yes, when do you perform HVE? | Simultaneously | 11 (39.3) | 5 (55.6) | 2 (66.7) |
|  |  | Sequentially | 9 (32.1) | 0 | 1 (33.3) |
|  |  | No answer because no PVE/HVE | 8 (28.6) | 4 (44.4) | 0 |
| 15 | Do you embolize segment IV? | Yes | 6 (21.4) | 0 | 1 (33.3) |
|  |  | No | 3 (10.7) | 1 (11.1) | 0 |
|  |  | When necessary | 19 (67.9) | 8 (88.9) | 2 (66.7) |
| 16 | Does non-target-embolization for the FLR play a significant and common role? | Yes | 2 (7.1) | 1 (11.1) | 0 |
|  |  | No | 24 (85.7) | 8 (88.9) | 3 (100) |
|  |  | No answer | 2 (7.1) | 0 | 0 |
| 17 | How long do you wait to resect after embolization? | 3 weeks | 3 (10.7) | 1 (11.1) | 1 (33.3) |
|  |  | 3-6 weeks | 20 (71.4) | 6 (66.7) | 2 (66.7) |
|  |  | >6 weeks | 4 (14.3) | 2 (22.2) | 0 |
|  |  | No answer | 1 (3.6) | 0 | 0 |
| 18 | Do you use the time for hypertrophy in CRLM to bridge with chemotherapy? | Yes, sometimes | 14 (50) | 5 (55.6) | 3 (100) |
|  |  | Yes, in most cases | 9 (32.1) | 3 (33.3) | 0 |
|  |  | Yes, always | 0 | 0 | 0 |
|  |  | No, never | 4 (14.3) | 1 (11.1) | 0 |
|  |  | No answer | 1 (3.6) | 0 | 0 |
| 19 | How many patients are resected after embolization? | <75% | 4 (14.3) | 0 | 0 |
|  |  | 76-85% | 11 (39.3) | 4 (44.4) | 1 (33.3) |
|  |  | 86-89% | 6 (21.4) | 2 (22.2) | 1 (33.3) |
|  |  | >90% | 6 (21.4) | 3 (33.3) | 1 (33.3) |
|  |  | No answer | 1 (3.6) | 0 | 0 |
| 20 | Patients, who do not get resected, … | ..mostly suffer a tumor progress | 16 (57.1) | 3 (33.3) | 1 (33.3) |
|  |  | ..mostly suffer from an insufficient FLR | 8 (28.6) | 4 (44.4) | 2 (66.7) |
|  |  | ..develop another contraindication for surgery or reject surgery. | 3 (10.7) | 2 (22.2) | 0 |
|  |  | No answer | 1 (3.6) | 0 | 0 |
| 21 | Do you see more postoperative complications after PVE/PHVE comparing to a major resection? | Yes | 3 (10.7) | 0 | 2 (66.7) |
|  |  | No | 25 (89.3) | 8 (88.9) | 1 (33.3) |
|  |  | No answer | 0 | 1 (11.1) | 0 |
| 22 | Do you consider SIRT as a suitable option for PVE? | Yes | 10 (35.7) | 3 (33.3) | 1 (33.3) |
|  |  | No | 18 (64.3) | 5 (55.6) | 2 (66.7) |
|  |  | No answer | 0 | 1 (11.1) | 0 |
| 23 | Is the combination of SIRT and PVE for hypertrophy a common procedure? | Yes | 6 (21.4) | 1 (11.1) | 0 |
|  |  | No | 22 (78.6) | 7 (77.8) | 3 (100) |
|  |  | No answer | 0 | 1 (11.1) | 0 |
| 24 | Do you test liver function preoperatively? | Yes, always | 9 (32.1) | 3 (33.3) | 1 (33.3) |
|  |  | Yes, if necessary | 12 (42.9) | 3 (33.3) | 2 (66.7) |
|  |  | No | 7 (25) | 3 (33.3) | 0 |
